# Obstetrics and perinatal outcomes of dichorionic twin pregnancy following ART compared with spontaneous pregnancy

**Published:** 2016-05

**Authors:** Leila Pourali, Sedigheh Ayati, Shahrzad Jelodar, Ahmadreza Zarifian, Mohammad Sobhan Sheikh Andalibi

**Affiliations:** 1 *Department of Obstetrics and Gynecology, Women's Health Research Center, Ghaem Hospital, School of Medicine, Mashhad University of Medical Sciences, Mashhad, Iran.*; 2 *Ghaem Hospital, School of Medicine, Mashhad University of Medical Sciences, Mashhad, Iran.*; 3 *Student Research Committee, School of Medicine, Mashhad University of Medical Sciences, Mashhad, Iran.*

**Keywords:** *Twin pregnancy*, *ART*, *Preeclampsia*, *Outcome*

## Abstract

**Introduction:**

Regarding to the recent advances in assisted reproductive techniques (ART), twin and multiple pregnancies have increased during past years.

**Objective:**

This study was performed to compare obstetrics and perinatal outcomes of dichorionic twin pregnancy following ART with spontaneous pregnancy.

**Materials and Methods:**

In this cross-sectional study which was performed in Ghaem Hospital, Mashhad University of Medical Sciences, 107 dichorionic twin pregnancy were enrolled in two groups: spontaneous group (n=96) and ART group (n=31). Basic criteria and obstetrics and neonatal outcomes information including demographic data, gestational age, mode of delivery, pregnancy complications (preeclampsia, gestational diabetes, preterm labor, and intrauterine growth retardation (IUGR), postpartum hemorrhage), neonatal outcomes (weight, first and fifth minute Apgar score, Neonatal Intensive Care Unit (NICU) admission, mortality, respiratory distress, and icterus) were recorded using a questionnaire.

**Results:**

Preterm labor, gestational diabetes, and preeclampsia were significantly higher in ART group compared to spontaneous pregnancy group. However, other factors such as anemia, IUGR, postpartum hemorrhage, and intrauterine fetal death (IUFD) were not significantly different between groups. There were no significant differences between groups in terms of neonatal outcomes (weight, 1^st^ and 5^th^ min Apgar score <7, NICU hospitalization, mortality, respiratory distress, and icterus).

**Conclusion:**

With regard of significantly higher poor outcomes such as preeclampsia, gestational diabetes and preterm labor in ART group, the couples should be aware of these potential risks before choosing ART.

## Introduction

Multiple gestation is one of the high-risk conditions that obstetricians and gynecologists are encountered. Moreover, twins are exposed to a significantly higher risk of intrauterine growth retardation (IUGR), complications like umbilical cord prolapse, placental abruption, placenta previa, asphyxia during labor, and birth trauma (1). During recent years, with progression of assisted reproductive techniques (ART) and increasing maternal age, the frequency of twin and multiple pregnancies has been significantly increased (2-4).

ART role in obstetric and neonatal outcomes of twin pregnancy has been investigated in several studies, however findings are controversial. Previous studies demonstrated that, twin pregnancy in ART patients have more complications than spontaneous pregnancy (4, 5). In the study performed by Moini *et al* which compared dichorionic twin pregnancy following fresh in vitro fertilization with spontaneously conceived dichorionic twin pregnancy in nulliparous women, concluded that obstetric outcomes were similar in both groups, but the risk of very preterm birth, NICU admission and perinatal mortality were higher in ART group (5).

However, the study of Anbazhaqan *et al* showed no significant difference in pregnancy complications and perinatal mortality between twin pregnancy following ART and spontaneous pregnancy (6). In a retrospective study by Geisler and colleagues maternal and neonatal outcomes of ART conceived and spontaneously conceived twin pregnancy were compared. They concluded that there was no significant difference in maternal complications, preterm birth, and NICU admission, but cesarean section, respiratory distress syndrome, and neonatal hypoglycemia were more common in ART group (7).

Current study was performed to compare obstetrics and perinatal outcomes of dichorionic twin pregnancy following ART with spontaneous pregnancy.

## Materials and methods

This cross-sectional study was done during 5 years from July 2009 to July 2014 at Ghaem Hospital, Mashhad, Iran. The study was approved by the Ethics Committee of Mashhad University of Medical Sciences. Written informed consent was obtained from all participants to confirm their agreement for participation in the study.

In total 127 women with dichorionic twin pregnancy who were categorized in two groups including spontaneous twin pregnancy (group A, n=96) and women with twin pregnancy following ART (long protocol) (group B, n=31) were enrolled. The study groups were consisted of women with spontaneous and ART induced dichorionic twin pregnancy which their chorionicities were mostly specified during first or early second trimester of pregnancy by ultrasonography which was confirmed by placental examination after delivery. 

The inclusion criteria were women with dichorionic and diamniotic twin pregnancy who were hospitalized due to labor. Exclusion criteria were the history of an underlying disease before gestation (e.g. overt diabetes, chronic hypertension, autoimmune diseases) monocorionic twin, and incomplete file data. The participants were selected by non-random convenience sampling method based on inclusion and exclusion criteria. 

Data were obtained by using questionnaires which were filled by research group. Questionnaire was filled by using of patients' file and included demographic features, gestational age with regard of last menstrual period or ultrasonography which was done at 10-20 wks of gestation, reason of developing twin pregnancy, mode of delivery, complications of pregnancy [preeclampsia (BP ≥140/90 mmHg + proteinuria ≥300 mg/24hr), gestational diabetes mellitus (GDM) (which is diagnosed for the first time during pregnancy), preterm labor (labor before 37 wks of gestation), IUGR (which was diagnosed by biometric ultrasonography)], gestational age at delivery, postpartum complications (postpartum hemorrhage which needs uterotonics or bimanual uterine massage or blood transfusion, uterine infection which was diagnosed by fever and prulant vaginal discharge or uterine tenderness), neonatal outcomes (weight, 1^st^ and 5^th^ min Apgar scores, NICU admission, mortality, respiratory distress, jaundice) and also maternal cigarette smoking or addiction. 

**Statistical analysis**

Data were analyzed by SPSS software (version 16, Chicago, USA). Qualitative and quantitative variables were described by central and distributive indexes and “Mean±SD” was used to explain them. In order to analyze qualitative variables, ^2^ test and Fisher's exact test were implemented while for analyzing quantitative variables, independent samples t-test was applied. P<0.05 was considered statistically significant.

## Results

The mean maternal age was not significantly different between group A and group B (27.1±4.7 vs. 28.9±5.0, respectively p=0.09). Statistical analysis showed that the number of gravidity was higher in group A compared to group B (2.2±1.2 vs. 1.3±0.5 respectively, p=0.002). However, we did not observe any association between gestational age and mode of delivery within groups (p=0.302, p=0.901 respectively). 

There were significant differences in frequency of preeclampsia onset, and premature uterine contractions (p=0.001, p=0.04, respectively) (Table I). Whereas, the prevalence of anemia, IUGR and post-partum hemorrhage and GDM were not significantly different between two groups (p=0.4, p=0.63, p=0.09, p=0.05, respectively).

After evaluation of perinatal parameters, we observed that birth weight, Apgar scores at 1^st^ and 5^th^ min were not significantly different between groups (p>0.05). Furthermore, the prevalence of ICU-admission, neonatal death, intrauterine fetal death, low birth weight, respiratory distress, congenital abnormality and icterus were not different between first and second twins in groups (Table II, III). We categorized 1^st^ and 5^th^ min Apgar scores into two groups including subject with Apgar score ≥7 and subject who had Apgar <7. The results suggest that Apgar score ˃7 at the 1^st^ and 5^th^ min after delivery were not different between groups (Table II).

**Table I:** Comparison of the frequency of maternal and perinatal outcomes between 2 groups

**Variables**	**Group A **	**Group B **	**p-value** *
Preeclampsia	13 (12.8)	11 (30.5)	0.007
Gestational diabetes	8 (7.9)	8 (22.2)	0.05
Anemia	5 (4.9)	3 (8.3)	0.4
IUGR	4 (3.9)	2 (5.5)	0.63
Post-partum hemorrhage	4 (4.2)	4 (12.9)	0.09**
Preterm labor	74 (77.1)	29 (93.5)	0.04
Total	96	31	

Data are presented as n (%).

IUGR: Intrauterine growth restriction

* *x* ^2^ test

** Fischer exact test

**Table II:** Comparison of first and fifth minutes Apgar scores < 7 between 2 groups

**Groups**		**Group A **	**Group B **	**p-value** *
Apgar at first minute < 7				
	First twin	22 (22.9)	6 (19.4)	0.67
	Second twin	23 (24.0)	7 (22.6)	0.87
Apgar at fifth minutes < 7				
	First twin	25 (26.0)	9 (29.0)	0.74
	Second twin	30 (31.2)	10 (32.3)	0.91
Total		100 (100.0)	32 (100.0)	

Data are presented as n (%).

* *x* ^2 ^test

**Table III:** Comparison of perinatal outcomes between 2 groups

**Variables **		**Group A **	**Group B **	**p-value** *
NICU Admission				
	First twin	42 (43.8)	18 (59.1)	0.16
	Second twin	43 (44.8)	17 (54.8)	0.33
Neonatal death				
	First twin	15 (16.9)	4 (13.3)	0.77
	Second twin	20 (22.2)	5 (16.7)	0.51
Intrauterine fetal death				
	First twin	7 (7.3)	1 (3.2)	0.67
	Second twin	6 (6.4)	1 (3.2)	0.99
LBW				
	First twin	86 (89.6)	29 (93.5)	0.72**
	Second twin	80 (83.2)	26 (83.9)	0.94
Respiratory distress				
	First twin	29 (29.2)	10 (31.2)	0.83
	Second twin	32 (32.6)	13 (42.9)	0.38
Congenital abnormality				
	First twin	10 (10.3)	5 (15.6)	0.52
	Second twin	13 (13.2)	3 (9.7)	0.75
Icterus				
	First twin	21 (21.9)	8 (25.8)	0.65
	Second twin	22 (22.9)	6 (19.4)	0.67

Data are presented as n (%).

LBW: low birth weight

* *x* ^2 ^test

** Fischer exact test

## Discussion

there was no significant difference in terms of delivery mode in two groups, but the rate of normal vaginal delivery was higher in spontaneous group and the rate of Cesarean section was higher in ART group (p=0.901) which was in concordance with the study of Caserta *et al* (8). Moreover, several studies have reported a significantly higher rate of Cesarean section delivery in ART group (7, 9-12). In these studies higher prevalence of Cesarean section in ART group can be due to poor obstetrical history in patients. Regarding the history of infertility and the childbirth importance in ART group, obstetricians prefer to perform Cesarean section. On the other hand, in the study done by Geisler and colleagues the rate of Cesarean section was higher in ART group, and the mean age of mothers in this group was about 4 yrs higher than spontaneous group (7). So, better myometrial function and elasticity of vaginal connective tissue in younger mother of spontaneous group and fewer tendencies of gynecologists to perform cesarean in this group could justify this difference.

In this present study, the prevalence of preeclampsia was significantly higher in ART group (p=0.007). Daniel *et al* also reported a significantly higher rate of preeclampsia in ART group, compared to spontaneous group (10). In the study done by Barat and colleagues, no significant difference was observed between ART and spontaneous groups regarding the preeclampsia prevalence, which can be due to their small sample size (13). In addition, Fan *et al* did not detect any significant difference for preeclampsia prevalence between groups (14). 

We found higher rate of GDM in ART group, but the difference was not significant (p=0.051), which is inconsistent with Fan *et al* and Montoya *et al* findings that reported no significant difference regarding GDM between groups, but the results of Barat *et al* and Caserta *et al* showed significantly higher rate of GDM in ART group (8, 13-15). These differences between the results of present study and the study of Barat *et al* regarding GDM might be due to racial differences. The prevalence of GDM and blood pressure is completely different in the eastern Asian race, compared to Iranian population.

We observed no significant difference regarding the prevalence of anemia and IUGR between groups (p=0.40, p=0.633 respectively), which is in line with the results of Fan *et al* and Montoya *et al* (14, 15). However, Daniel *et al* and Barat *et al* found significantly higher prevalence of IUGR in ART group, compared to spontaneous group (10, 13). The larger sample size in the latter two studies might be a reason for these differences.

Two groups of this study showed no significant difference regarding postpartum hemorrhage (p=0.099) which is similar with Fan *et al* findings (15). There is a significant difference between two groups regarding premature uterine contractions (p=0.04). This is in line with several previous studies in which the rate of premature uterine contractions was higher in ART group (7, 8). However, other studies reported similar rate of premature delivery between two groups, which might be due to smaller sample size of these studies (7, 11, 14, 15). Additionally, Fan *et al* have mentioned delivery after 28^th^ wk as one of their inclusion criteria, which can cause lower rate of premature deliveries in their study (14). Racial differences are also a cause of difference between the results of Zaib-un-Nisa *et al* and our results (11).

We also assessed neonatal outcomes in present study. No significant difference was observed considering neonatal weight and the prevalence of low birth weight (LBW) between groups (p=0.72 for first twin, p=0.94 for second twin). This is in line with many previous studies (6, 11). Nevertheless, three other studies found lower mean neonatal weight and higher rate of LBW in ART group compared to spontaneous group. It may be due to significantly higher rate of premature delivery in ART group compared to spontaneous group in all three mentioned studies (2, 10, 12).

In the present study, no significant difference was found in terms of 1^st^ and 5^th^ min Apgar scores between groups (1^st^ min Apgar score for first twin (p=0.67) and for second twin (p=0.87)) [5^th^ min Apgar score for first twin (p=0.74) and for second twin (p=0.91)]. The studies done by Andrigasevic *et al*, Caserta *et al* and Fan *et al* also reported the same findings (8, 12, 14). However, Daniel *et al* found significantly higher rate of low 5^th^ min Apgar scores (<7) in ART group compared to spontaneous group (10). This might be due to higher rate of premature delivery and consequent LBW, and larger sample size in their study.

In our study, the rate of NICU admission was not significantly different between groups (p=0.16 for first twin, and p=0.33 for second twin), but several studies have found higher rates of NICU admission in ART group compared to spontaneous group (8, 10, 15). This difference might be due to higher rate of premature delivery, LBW, and low Apgar scores or their larger sample size in ART group compared to spontaneous group in mentioned studies. Although the frequency of neonatal death was higher in spontaneous group, there was no significant difference between groups regarding this complication (p=0.77 for first twin, and p=0.51 for second twin). This is similar to the results of Andrigasevic *et al* and Geisler *et al *(7, 12). 

We observed no significant difference between two groups regarding intrauterine fetal death (IUFD) for both first and second twins (p=0.67, p=0.99 respectively), which is in line with the studies of Barat *et al* and Fan *et al* (13, 14). Other studies have found significantly higher rate of IUFD in ART group because of higher incidence of infection and prematurity in ART group (8).

Other neonatal complications such as neonatal respiratory distress, icterus, and congenital anomalies were not significantly different between groups, which the results are similar to studies done by Fan *et al* and Caserta *et al* (8, 14). However, some other studies reported a higher rate of respiratory distress in ART group, which may be due to the higher rate of Cesarean section in ART group in these studies (7, 9). 

The limitations of this study were lack of evaluating the cause of infertility and ART type (ICSI or IVF) in ART group, missing and unavailable data files that led to the exclusion of some patients from study. The strengths of this study included the matching for age between groups to eliminate its confounding effect. Moreover, we considered the chorionicity status of twins and included dichorionic diamniotic twin pregnancy which was confirmed by ultrasonography. We also investigated addiction and smoking in participants, which could be considered in results interpretation.

It is recommended that more prospective studies with larger sample size be performed with regard of ART type and cause of infertility to obtain more accurate results.

## Conclusion

According to higher prevalence of maternal complications such as GDM, preeclampsia, and premature delivery in ART-induced twin pregnancy, preconception counseling is strongly suggested in couples undergoing ART. In addition, after the conscious selection of ART, the patient should undergo accurate prenatal care and screening for early diagnosis and treatment of possible complications of twin pregnancy.

## Note

***This article extracted from M.D. thesis. (Shahrzad Jelodar)***
